# *Enterococcus faecalis* Septicemia and Vertebral Osteomyelitis after Transrectal Ultrasound Guided Biopsy of the Prostate

**DOI:** 10.1155/2015/159387

**Published:** 2015-11-22

**Authors:** Ayaz Virji, Lucio R. Minces, Zargham Abbass

**Affiliations:** ^1^Johnson Memorial Health Services, 1282 Walnut Street, Dawson, MN 56232, USA; ^2^Infectious Disease, Affiliated Community Medical Centers, 101 Willmar Avenue SW, Willmar, MN 56201, USA

## Abstract

Transrectal ultrasound guided prostate biopsy (TRUS) has rarely been associated with disseminated infection, yet the occurrence appears to be increasing. Resistance to fluoroquinolones, the most commonly used prophylaxis, is one of the likely causes, with* Escherichia coli* being the most commonly reported cause of these infections. Herein we present what is, to our knowledge, the first case of* Enterococcus faecalis* septicemia and vertebral osteomyelitis after TRUS. Previously reported cases of this condition are referenced also.

## 1. Introduction

Transrectal ultrasound guided prostate biopsy (TRUS) carries a risk for urinary as well as disseminated infection. These risks were historically low, given the use of oral ciprofloxacin as prophylaxis [1]. For various reasons, including increased resistance to quinolones, the incidence of post-TRUS infection is on the rise [1, 2]. Most of the reported cases are related to* Escherichia coli*, as summarized in this report. We present the case of a patient who developed disseminated infection with* Enterococcus faecalis* after TRUS.

## 2. Case Presentation

A 77-year-old male underwent TRUS due to an elevated PSA level of 15.8 ng/mL. Oral ciprofloxacin 500 mg twice a day, for 3 days, was used for prophylaxis, starting the day before the procedure. His biopsy result was benign. Three weeks later, he presented to our clinic complaining of acute onset back pain and nausea; the pain was sharp, constant, and worse with movement. He had no associated hematuria or dysuria. His past medical history is relevant for well-controlled moderate persistent asthma, on daily prednisone (5 mg/day); he had been on this medication for at least several months prior to this event. An initial roentography of the lumbar spine showed degenerative joint disease with no acute abnormality, and urinalysis was negative for pyuria or hematuria. Complete blood count showed leukocytosis of 16,000 cells/mcl. The patient was admitted and empirically started on intravenous ceftriaxone one gr every 24 hours. A noncontrast enhanced magnetic resonance imaging (MRI) of the lumbar spine ordered the day after admission (because of his persistent back pain) showed degenerative changes with mild central and foraminal stenosis, without acute infection. The patient continued to have severe back pain and fever. A computed tomography of the pelvis demonstrated an enlarged and heterogenous prostate and fluid in the right inguinal canal; these were nonspecific findings and not thought to be a nidus of infection. Blood cultures obtained at admission initially grew out Gram positive cocci in pairs, and his antibiotics were changed to vancomycin for a goal trough of 15–20 mcg/mL, pending susceptibilities. This organism was later identified as* Enterococcus faecalis*. Once susceptibilities were available, he was switched to ampicillin 12 gr a day. A repeat blood culture after three days on appropriate therapy (the organism was sensitive both to vancomycin and ampicillin, as well as synergistic aminoglycosides) remained positive. Based on the persistent bacteremia, the patient was started on synergistic dose gentamicin (one mg/kg every 8 hours) as well, and transferred to a tertiary care center.

**Figure 1:**
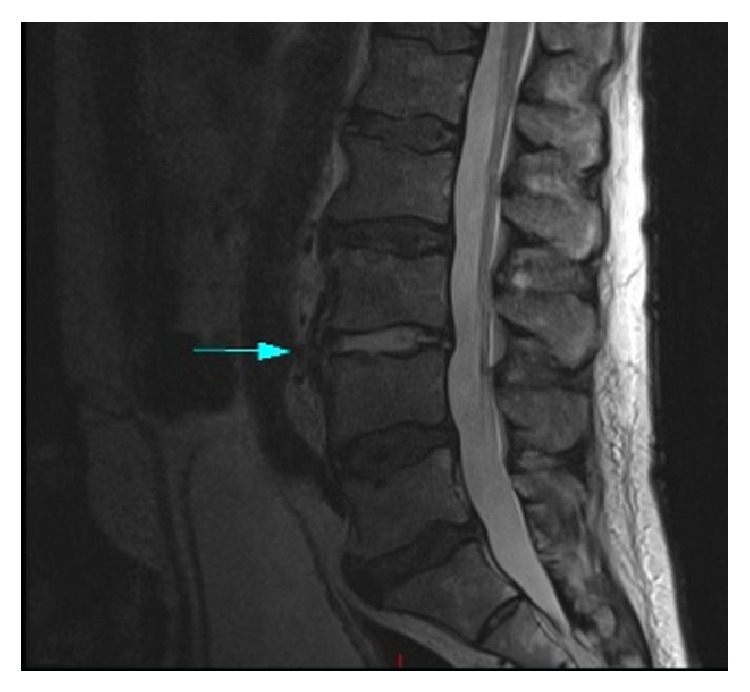


Transesophageal echocardiogram was negative for infective endocarditis. A repeat, contrast enhanced MRI of the lumbar spine showed phlegmonous changes suspicious for discitis and osteomyelitis at the L3-L4 segment (Figure 1). He was diagnosed with vertebral osteomyelitis following TRUS. The patient responded well to a six-week course of IV ampicillin and gentamicin, with resolution of his symptoms. The decision to complete dual therapy versus monotherapy with ampicillin was based on the high grade bacteremia noted at initial presentation and the lack of bactericidal activity of ampicillin for enterococci.

## 3. Discussion

TRUS is a common urological procedure used to differentiate prostate cancer from benign prostatic hyperplasia. Hospitalization for urologic complications following TRUS has increased fourfold from 1996 (1.0%) to 2005 (4.1%) [2]. Common complications from the procedure include transient hematuria, hematospermia, rectal bleeding, and urinary tract infection. Rare complications include sepsis and vertebral osteomyelitis. To our knowledge, there have been a total of five reported cases, including ours, of vertebral osteomyelitis following TRUS [3–6]. A plexus of veins known as Batson's plexus drains the lower bladder and prostate and communicates with the vertebral venous circulation. This plexus lacks traditional valves and is believed to be the mode of entry for metastatic cancer and infections from the lower urinary tract to the spine [7].

The rarity of this condition, coupled with an original MRI lacking evidence of infectious etiology, delayed our discovery of the source for the patient's sepsis. It was only after a repeat positive blood culture and repeat MRI with contrast that vertebral osteomyelitis was confirmed. The patient's initial presentation of symptoms included fever, leukocytosis, and back pain and occurred approximately three weeks after his TRUS. Despite the intuitive notion that such an adverse reaction should appear in the more immediate postprocedure timeframe, the onset of vertebral osteomyelitis after TRUS is more insidious and progressive. This patient was on chronic prednisone. The role of this medication on his infection is unclear, but likely irrelevant, considering he was on low dose, and the bacteremia was generated by a disruption of the mucosal barrier (biopsy). The other cases reported in the literature that we reviewed did not mention immunosuppression in these patients as a potential cause (or risk factor).

Infectious complications of prostate biopsy procedures are on the rise and may be due (in part) to patterns of antimicrobial resistance [8], as evidenced in two of the referenced cases [4, 5]. In our particular case, it is to be noted that ciprofloxacin, which was used for prophylaxis, might not have been active against* Enterococcus*. While it was sensitive to it, this quinolone is known to offer variable activity against this organism [9]. Given that vertebral osteomyelitis can occur up to one month after TRUS, suspicion and early recognition are essential for timely management.

## References

[1] Williamson D. A., Roberts S. A., Paterson D. L. (2012). *Escherichia coli* bloodstream infection after transrectal ultrasound-guided prostate biopsy: implications of fluoroquinolone-resistant sequence type 131 as a major causative pathogen. *Clinical Infectious Diseases*.

[2] Nam R. K., Saskin R., Lee Y. (2013). Increasing hospital admission rates for urological complications after transrectal ultrasound guided prostate biopsy. *Journal of Urology*.

[3] Rajgopal R., Wang Y., Faber K. J., Izawa J. I. (2012). Vertebral osteomyelitis following transrectal ultrasound-guided biopsy of the prostate. *Journal of the Canadian Urological Association*.

[4] Assimacopoulos A., Johnston B., Clabots C., Johnson J. R. (2012). Post-prostate biopsy infection with *Escherichia coli* ST131 leading to epididymo-orchitis and meningitis caused by gram-negative bacilli. *Journal of Clinical Microbiology*.

[5] Roberts M. J., Parambi A., Barrett L. (2013). Multifocal abscesses due to multiresistant *Escherichia coli* after transrectal ultrasound-guided prostate biopsy. *Medical Journal of Australia*.

[6] Zimmerli W. (2010). Vertebral osteomyelitis. *The New England Journal of Medicine*.

[7] Onuigbo W. I. B. (1975). Batson's theory of vertebral venous metastasis: a review. *Oncology*.

[8] Johnson J. R., Polgreen P. M., Beekmann S. E. (2015). Transrectal prostate biopsy-associated prophylaxis and infection complications: report of a query to the emerging infections network of the Infectious Diseases Society of America. *Open Forum Infectious Diseases*.

[9] Perry J. D., Ford M., Gould F. K. (1994). Susceptibility of enterococci to ciprofloxacin. *Journal of Antimicrobial Chemotherapy*.

